# Amalgam as a Filling-Material

**Published:** 1893-08

**Authors:** Edward Page

**Affiliations:** Charlestown, Mass.


### AMALGAM AS A FILLING-MATERIAL.[1]

BY EDWARD PAGE, M.D., D.M.D., CHARLESTOWN, MASS.

Mr. President and Members of the Society,—To go into the subject thoroughly in relation to its history, manufacture, and use would take more time than I or you would desire to give to it, therefore I must be very brief; but I presume I shall say enough to excite a reasonable amount of discussion; if not, then I shall consider this paper somewhat of a failure.

I have used various dental alloys during the last thirty years, and have been a manufacturer for seven years. Twenty-five years ago I used gold almost exclusively for filling teeth. Since then I

---

[1] Read before the Massachusetts Dental Society at its annual meeting, June 8, 1893.

have gradually used more amalgam, cement, and gutta percha, and less gold, until at the present time I am using only about one-third as much gold as I did years ago in the same number of operations. I use amalgam because I know it is the best material for the case in hand, and not because some one has dogmatically said it was good or bad without giving any facts or reason for the opinion.

The first record of its use in this country was in the year 1833, sixty years ago. Up to the year 1850 the amalgam used was unsatisfactory on account of a lack of experience in its manufacture and use, and the best results were not always accomplished. In 1855, Dr. Elisha Townsend recommended an alloy composed of four parts silver and five parts tin. This, or a similar kind, has been in use to the present time. All good dental alloys are composed more or less of the following metals: silver, tin, copper, gold, platinum, and zinc. Some alloys contain silver and tin only, others silver, tin, and copper, still another kind silver, tin, copper, gold, platinum, and zinc. Thus an almost endless variety can be made from these six metals by using two or more kinds in different proportions. Silver is the most important metal used in making dental alloy. In fact, without silver, dental amalgam would be nearly valueless. The general average of its use is more than any other metal except tin. It is essential for making a hard setting amalgam and for maintaining the integrity of its structure. Tin is second in importance in making dental alloys. It has no chemical affinity for mercury, but it is a medium of solid vehicle in which the silver molecules are mixed mechanically and appropriately for amalgam purposes. Copper is frequently used, from one to ten per cent., generally about five per cent. It increases plasticity and makes a harder amalgam. but slightly increases shrinkage, and may cause an inflow of the fluids of the mouth between the surfaces of the fillings adjacent to the walls of the cavity. Consequently this space is filled with a black sulphide of silver and copper discoloration, which hardens the dentine and wonderfully preserves the tooth substance. On this account some dentists claim that an alloy that shrinks is the best, and I think it appears to be reasonable.

Gold is not commonly used in dental alloys. It does not increase edge strength, hardness, or toughness. The only benefit is a slight increased plasticity and quick setting, and it makes a smoother filling; consequently it controls shrinkage. Zinc would produce a similar result in a much smaller quantity, with the exception of decreasing the plasticity instead of slightly increasing it, as does gold, and also retard black discoloration, as gold does not. Therefore zinc is more valuable than gold. It is a very useful metal on several accounts. It controls shrinkage, is quick setting, and hinders black discolorations. Twenty out of the fifty-four alloys on the market contain zinc.

Platinum is of no benefit to amalgam; it increases brittleness in alloys and renders them liable to crumble in proportion to the amount used. It may in a small degree produce quick setting, consequently prevents shrinkage, but this could be accomplished by using zinc or gold, without the injurious effects of platinum.

A great many reasons have been given why fresh-cut alloy is the best, but if all alloys are mixed according to the following rules, it will make but little difference whether fresh cut, coarse, or fine. Use a Wedgwood mortar instead of by the hand. Prepare a teaspoonful of hydrochloric acid to a pint of water. Keep this in a large bottle; pour a sufficient quantity into the mortar, add the alloy, stir with a pestle, add the mercury, amalgamate; then wash in plain water; dry out the moisture as much as desired before using.

By this method the acid will cleanse the black oxide from each particle of alloy. Amalgam treated in this way will set quicker and stronger, because there is not so much discoloration to interfere with the chemical affinity between the silver and mercury, also with less tendency to discoloration because of the decreased amount left in the amalgam mass. Therefore silver and copper, but principally silver, is the cause of the discoloration on amalgam fillings. Although silver has but little affinity for oxygen it unites with the allied element, sulphur, more readily than any other metal. The tarnish on amalgam filling is caused by the formation of a thin film of sulphide from the traces of sulphur compounds usually present in the air. Matches carried in the same pocket with silver coin will illustrate this point. Consequently the alloy that contains the smallest amount of silver and copper and the largest amount of tin, zinc, and gold will make the lightest-colored fillings.

A great many dentists object to using mercury enough to make an amalgam plastic, for the reason that in pressing out the surplus a large percentage of the metals will be carried with it. This has no real foundation in fact, as experiments have proved that only one five-thousandth of one per cent. of gold, and the same amount of silver, and only a trace of platinum, too small for estimation, is pressed out of a mix of dental amalgam containing four hundred and eighty grains. This is so small that there can be no objection to using all the mercury required to make a mix sufficiently plastic.

No history of dental amalgam would be complete without reference to the amalgam war which commenced in this country in the year 1840, and was carried on within the ranks of the dental profession. The opposition to the use of amalgam had no scientific basis, as nearly all who opposed it had, in the language of one noted opponent, "never touched the nasty stuff." The opposition to its use did not realize that it would be well to prove all things and hold fast to that which is good. In this case, like all others, the agitation of thought proved to be the beginning of wisdom. The same opposition was active when vulcanite rubber was introduced into the profession as a base for artificial dentures, and like amalgam has won its way to popular favor by its intrinsic value. A ten years' warfare was waged against its use, the most bitter and unreasonable that has ever disturbed the harmony of the dental profession, and practically ended in 1850, so far as any dental society's authority or jurisdiction was concerned, by the adoption of the following resolution: "*Resolved*, That the several resolutions adopted by the American Society of Dental Surgeons at the annual meetings held in 1845 and 1846, having the effect of enforcing subscription to the protest and pledge against the use of amalgam and mineral paste for filling teeth, be and the same are hereby rescinded and repealed." Since then its merits have gradually become appreciated, until, as a rule, dentists, including nearly all the best operators, use it when in their opinion its use is indicated.

A tooth is like a sick person, it should receive such treatment as would be best for that particular case. Gold is sometimes the best material to use and sometimes it is the poorest, and so of amalgam, cement, and gutta-percha; discrimination in filling-materials is of the utmost importance in the treatment of diseased teeth. Not long since a dentist, who is not a member of this Society, but has an office in a prominent street on the Back Bay, said to me, "I use nothing but gold in filling teeth." In the light of the present day, educationally and conscientiously considered, what a statement! Can such a man be honest and intelligent? I have my opinion, you are at liberty to have yours.

The question, What shall we use for filling teeth? is not new, but is as important as ever. One noted dentist said, several years ago, "In proportion as a tooth needs saving, gold is the poorest material we can use." This statement I believe to be correct, and I have practised in accordance with it for a number of years.

Amalgam is unique as a filling-material. Large crown cavities in molars can be partially filled with cement or gutta-percha, and finished with amalgam, or it can be used in filling cavities difficult of access. It is also very useful where the cavity goes below the gum on the buccal or approximal surfaces of molars. The cavity can be partially filled with amalgam and finished with gold. Likewise large crown cavities where it would be unsafe to fill with gold or alloy.

Dr. Clapp, a member of this Society, has given clinics before several societies to illustrate his method of combination fillings. Fill the bottom of the cavity with cement, cover this with alloy, then finish with Steurer's gold. Thus we have the benefit of three filling-materials. The cement is a poor conductor of heat and cold, and keeps the tooth comfortable. The alloy is easily packed over the cement. The cavity is then finished with gold before the alloy hardens, and thereby it is firmly attached to the alloy. Thus a satisfactory appearing filling is produced with all the good qualities of each material, and is of more real value than could be made with either alone.

Dead teeth, or teeth badly decayed, can be filled or built on with amalgam so as to make them useful for years. Amalgam in such cases is almost indispensable, and as one dentist has said, and I think truthfully, "a good amalgam filling is better than a poor gold filling." A few well-meaning people really believe that amalgam fillings are dangerous, notwithstanding amalgam as a filling-material has been used for sixty years in this country and by millions of people with no serious results so far as any scientific proof is concerned. In the first place, we cannot reason a person out of that which they have never been reasoned into. Therefore they that have accepted a dogma in relation to medical practice cannot be very easily reasoned out of it. A few persons, principally on the Back Bay, who practice homœopathy appear to take the responsibility to dictate to the profession of dentistry what shall be used as a filling-material. This profession stands second to no other in relation to education, scientific investigation, and painstaking research into the causes of disease. Who are the persons who object to amalgam? Are they our scientists, men who have made a study of nature's laws, those who are familiar with the microscope, chemical and quantitive analysis? As a rule, I think not, but are those who have accepted some one's dogmatical say so, and are like the Scotchman, who said, "I am open to conviction, but where is the man who can convince me?" I desire to approach this subject of amalgam in an impartial spirit, without fear or prejudice, as I am a student seeking for truth in every department of life.

I know very well that there are and always have been a few who have objected to its use for filling teeth. Now, if there is any serious or valid reason why we should not use it, I would be glad to know it, not believe it, but have scientific evidence given supported by facts so abundant that there could be no reasonable doubt about it; then I for one would not make or use it in my practice. A patient once said to me, "I suppose gold is the best filling for teeth." I replied, "Sometimes it is, and sometimes it is the poorest." If we could have but one kind, either gold or amalgam, for filling teeth, I would say, abolish gold and retain amalgam as the best. I would say about gold as a filling-material as Dr. Holmes said about medicine, if all the gold were thrown into the sea it would be better for the people and perhaps worse for the fishes.

I know of dentists who say they use more gold in filling teeth than accords with their judgment because their patients demand it. Men may theorize as much as they please, but every case is settled by an extensive practical demonstration. Dr. Lardner, a scientist, gave a lecture in Boston many years ago to prove theoretically that it would be impossible for a steamer to cross the Atlantic. At the same time a steamer was crossing and arrived safe and sound in due time. Scientific theories without practical proof have thus many times come to naught, and so with those regarding amalgam.

A few physicians say amalgam in teeth is unsafe and an obstacle in the way of curing disease, when it has been used more or less for sixty years in every civilized country with no difficulty, and has been found to be no obstacle in the treatment of disease.

Some dentists have noticed an irritation on the mucous membrane opposite an amalgam filling. It seems plain that this inflammation could be produced by jagged edges of the filling. Dr. Foster Flagg says he has frequent complaints of this kind, and cases have been sent to him to illustrate the evil effects of amalgam fillings, and he has invariably cured the patients without removing the fillings. The difficulty is a lack of thoroughness. Because the filling is an amalgam do not fail to do the best work, prepare the cavity as perfectly as for a gold filling, see that your alloy is well amalgamated, avoid all slovenly work, leave no mass of the material overhanging the cavity at the cervical margin to irritate the soft tissues, or neglect to polish the filling when hard. If there is faithfulness in these particulars a good filling will be the result, and no cause of complaint will be made of irritated parts near an amalgam filling. Success in all callings and all operations depends on thoroughness. Success or failure is due to the operator more than the material. There is no trouble in finding amalgam fillings that have done good service for twenty or thirty years, showing that amalgam has virtues that no other material possesses. Dr. Bonwill, who has had a wide experience with filling-materials, said a year or two ago, "I am filling more teeth with amalgam than ever before." From what I can learn I think this is true of the profession throughout the country. When a physician looks into a patient's mouth and says, "That is bad," the patient asks, "What is bad?" and the doctor replies, "Amalgam fillings in your teeth," I would say that such indiscriminate and wholesale statements are wrong and have no foundation in fact, and are as unscientific and unprofessional as the statement made by a doctor in his experience with yellow fever, to the effect that a certain remedy he had used would cure a Frenchman but would kill a Scotchman.

Perhaps there may be rare cases where a person would be unpleasantly affected by an amalgam filling in the teeth, but so rare that scarcely a case would be found in an ordinary dental or medical practice of forty years. If this be so, is it worthy of our attention, or can it have any bearing on our regular practice? In all cases where the physician should advise the removal of amalgam fillings on account of being harmful the diagnosis ought to be doubted at once, and a consultation called for, with a demand for the scientific reasons for such diagnosis. If dentists would insist upon this course we would soon hear but little more in regard to the bad effects of amalgam fillings.

I recently called upon a homœopathic physician in Boston who is noted for his opposition to this material. He did not wish to make a written statement or give any reasons for his dislike of amalgam filllings, but stated in a general way that he had cases where he failed to effect a cure on account of amalgam fillings in the patient's teeth, and when these were removed or the teeth extracted the disease was cured without any serious difficulty. He mentioned one case where he treated a young lady for an inflammation in the mouth unsuccessfully. He noticed, as he said, a stub built up with amalgam stuff, and ordered the tooth extracted, after which he found no difficulty in curing the lesion. He cited this to prove that amalgam fillings were harmful.

To my mind this is a very simple case. Probably no more so than nearly or quite all those reported as to the bad effects of amalgam fillings, and can be explained thus: the diagnosis was incorrect.

Evidently the tooth was dead. The swelling was a fistula caused by an abscess, or, if the tooth was not devitalized, may have been caused by a jagged edge of the filling overhanging the cavity. In either case if the tooth were extracted the inflamed surface would get well without any treatment whatever. I also had an interview lately with another homœopathic physician of large practice. He said in all this time he had not seen a case where he thought amalgam fillings were in any way harmful, and that he looked upon certain physicians on the Back Bay who object to amalgam as cranks, and I believe from what I learn that this view is entertained by almost all medical practitioners from every school of medical practice. Therefore we should insist that no amalgam fillings shall be removed or teeth extracted by the order or advice of any physician without professional consultation with the patient's dentist, and then only by mutual consent.

Dr. S. B. Palmer, of Syracuse, N. Y., who has had a large experience with amalgam, says, "I have no facts or proof that leads me to think amalgam as a filling is injurious. I know of cases where all amalgam fillings have been removed without benefit, and the physician who advised it being done felt the reaction through the disappointed patient."

A society of scientific gentlemen in Europe a number of years ago tested vulcanite rubber in saliva under all degrees of heat likely to exist in the mouth to discover if mercury could be detected in this fluid, but no evidence of it could be found. In the language of Professor Mayer, I would say, "I doubt as to whether we can speak of any physiological action of amalgam beyond the very small border line of dentine into which the products of oxidation and sulphurization penetrate. That a small amount of mercury may be dissolved from amalgam, not through the dental tubuli, but from their surfaces, aided by the process of mastication, seems entirely reasonable and probable, but the quantity is so small that it cannot be well ascertained by weight in the laboratory."

A great many persons who have been more or less affected with disease have been told that their amalgam fillings must be taken out before a cure could be accomplished. The fillings were not taken out, and they got well with no unusual delay or unlooked-for difficulty. I will mention one case of many. A young lady called on a dentist of my acquaintance and a member of this Society and requested him to remove all her amalgam fillings, as her mouth was in a diseased condition. A noted homœopathic physician had urged that this be done. She was advised to consult another doctor, and did so. He made no objection to amalgam, and treated her, with complete recovery in two weeks, without having her fillings removed.

The following case came under the observation of a dentist who is a member of this Society. A lady under treatment by a very prominent homœopathic physician was also a patient of this dentist, who, knowing that her physician would not be in favor of amalgam, filled her teeth with cement and gutta-percha. Her medical treatment did her no good, and after a while she went to New York and other places, during which time she was under the treatment of her physician. While she was away she had her cement and gutta-percha fillings replaced with amalgam. Soon after she began to improve in health until she apparently was in good condition. Now, it would be just as logical to say that homœopathic remedies would do no good until the patient had her teeth filled with amalgam as it is that disease cannot be cured until amalgam fillings are taken out. Such cases only show how easily one could jump to a conclusion without any scientific reason, and dogmatically decide a question without a thought.

A lady wished to have an amalgam filling removed, as it made her sick. The dentist made use of a little Yankee wit, which might be considered questionable. He said to her, "I will replace this amalgam filling with one of silver," and did so, and she had no more trouble with it.

Early in life Sir Humphry Davy assisted Dr. Beddoes in his experiments on the inhalation of nitrous oxide. Dr. Beddoes having inferred that the oxide must be a specific for palsy, a patient was selected for trial and placed under the care of Davy.

Previously to administering the gas, Davy inserted a small thermometer under the tongue of the patient to ascertain the temperature. The paralytic man, wholly ignorant of the process to which he was to submit, but deeply impressed by Dr. Beddoes with the certainty of its success, no sooner felt the thermometer behind his teeth than he concluded the talisman was in operation, and in a burst of enthusiasm declared that he already experienced the effects of its benign influence throughout his whole body.

The opportunity was too tempting to be lost. Davy did nothing more, but desired his patient to return on the following day. The same ceremony was repeated, the same result followed, and at the end of a fortnight he was dismissed cured, no remedy of any kind, except the thermometer, having been used.

One person cannot eat cheese, another cannot comfortably stay in a room with an apple, coffee, honey, or a cat.

Queen Anne would faint at the sight of a rose. In order to test this case, an artificial rose was shown her and she fainted just the same, which proves that as a man thinketh so he is.

This condition is the peculiar idiosyncrasy of some individuals. When a person is under the control of a dogma in relation to medical practice or anything else, science, reason, or facts will have but little effect to change his mind, but education in all its branches will do it in time, however slow.

This is also true in regard to amalgam. What is the sensible and scientific practice relating to the cure of disease? It is to use any remedy that will help the patient. This certainly is reasonable. Any substance or law is open to us all to use as a remedy for the sick. If a few doctors find their remedies incompatible with amalgam in the teeth, so much the worse for their remedies, and they should be given up as unworthy the name.

I do not blame the few medical practitioners for their opposition to amalgam fillings, as they evidently do so for conscience' sake. I should do the same if I were satisfied that amalgam as a filling-material was harmful, but because a person is honest that is no proof that he is wise. It is not sufficient that a doctor be honest. He ought also to be intelligent. Either alone would lead to trouble. If both go together, we may expect the best results.

Therefore to object to amalgam in order to have the opinion respected, there must be given an honest and intelligent reason.

We who use amalgam are satisfied that it is useful and harmless. Therefore let those few persons who object to it on any ground give us and the world some good plain scientifie reasons to prove it unfit to use in our practice, but for the few to denounce it as incompatible with a healthy condition of the mouth is absurd, and unworthy the notice of our profession. The demand of our time is, What do you know? instead of How much do you believe?

Now, in conclusion, let us all be reasonable. If there is any one who can show cause why amalgam should not be used as a filling material, let us calmly listen to substantial proof and scientific demonstration; but if no such reasons can be given, then, in view of these facts, let us continue to use it when its use is indicated by our judgment.

